# Geochronological results from the Zhela Formation volcanics of the Tethyan Himalaya and their implications for the breakup of eastern Gondwana

**DOI:** 10.1038/s41598-023-47268-5

**Published:** 2023-11-16

**Authors:** Jiacheng Liang, Weiwei Bian, Xianwei Jiao, Wenxiao Peng, Jiahui Ma, Suo Wang, Yiming Ma, Shihong Zhang, Huaichun Wu, Haiyan Li, Yuruo Shi, Tianshui Yang

**Affiliations:** 1 State Key Laboratory of Biogeology and Environmental Geology, China University of Geosciences, Beijing, 100083 China; 2 School of Earth Sciences and Resources, China University of Geosciences, Beijing, 100083 China; 3 School of Earth Sciences and Resources, China University of Geosciences, Wuhan, 430074 China; 4 Beijing SHRIMP Center, Institute of Geology, Chinese Academy of Geological Sciences, Beijing, 100037 China

**Keywords:** Solid Earth sciences, Geochemistry, Palaeomagnetism, Tectonics

## Abstract

The relationship between the Kerguelen mantle plume and the breakup of eastern Gondwana is still debated. The new Zircon SHRIMP U–Pb dating of 139.9 ± 4.6 Ma, as well as previous ages from the Zhela Formation volcanic rocks in the Tethyan Himalaya, show that the studied Zhela Formation volcanic rocks formed during the Late Jurassic-Early Cretaceous, rather than the Middle Jurassic. The calculated volume of the Comei-Bunbury igneous rocks is ~ 114,250 km^3^, which is compatible with the large igneous provinces and, consequently, the typical mantle plume models. The new date results, along with existing dates, show that the volcanism attributed to the Kerguelen mantle plume in the Tethyan Himalaya ranges from ca.147 Ma to ca.124 Ma, with two peaks at approximately 141 Ma and 133 Ma. This new finding, together with geochemical and palaeomagnetic data obtained from the Comei-Bunbury igneous rocks, indicate that the Kerguelen mantle plume contributed significantly to the breakup of eastern Gondwana and that eastern Gondwana first disintegrated and dispersed at ca.147 Ma, the Indian plate separated completely from the eastern Gondwana before ca.125 Ma.

## Introduction

**Figure 1:**
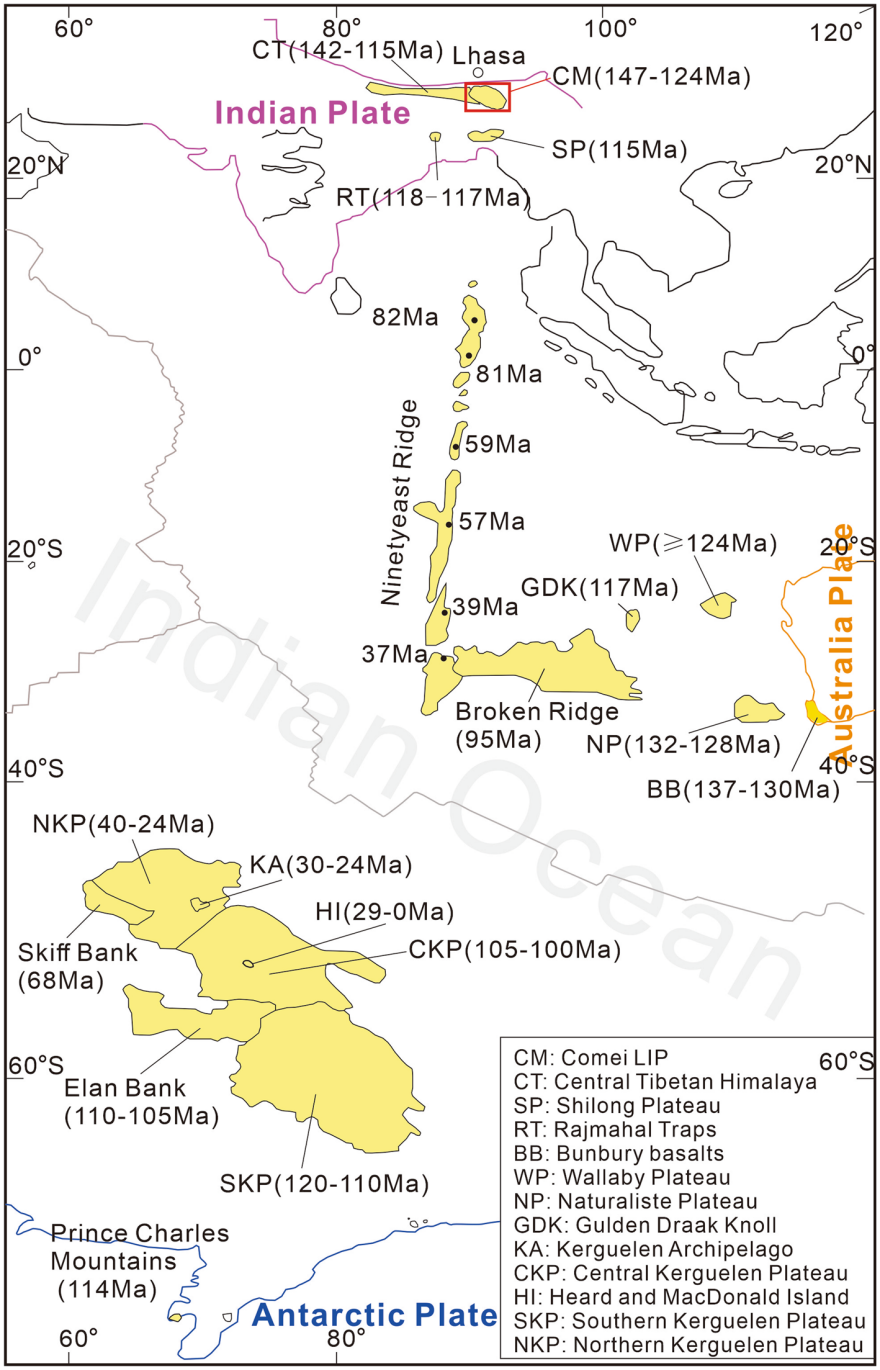
Temporal and spatial distribution of products related to the Kerguelen mantle plume (after Zhu et al.^9^).

Large igneous provinces (LIPs) are magmatic provinces with areal extents > 100,000 km^2^, igneous volumes > 100,000 km^3^, and overall eruptive durations of around 50 Myr, including vigorous eruptive pulse(s) of approximately 1–5 Myr, during which a significant proportion (> 75%) of the entire volume of the LIP is emplaced^1^. LIPs have been intensively investigated due to their close spatial and temporal coincidence with mass extinctions, regional-scale uplift, continental rifting, and their significant impact on global palaeoclimate, palaeoenvironment, and the evolution of life^2–6^. From the Late Jurassic to the present, the long-lived Kerguelen mantle plume has been characterized by massive magma output, leading to the formation of the Kerguelen LIP and being connected to the breakup of eastern Gondwana^7–11^. Igneous rocks forming the Kerguelen LIP include Comei (eastern Tethyan Himalaya), Bunbury (southwestern Australia), Rajmahal (northeast India), Southern-Central-Northern Kerguelen Plateau, Broken Ridge, and Ninetyeast Ridge (Fig. 1).

The Rajmahal Traps in northeast India and the contemporaneous lavas in the southern Kerguelen Plateau (ca. 120–110 Ma) were once widely accepted as the first manifestation of the Kerguelen mantle plume^8^. However, different investigators have different views regarding the older igneous rocks on the circum-eastern Gondwana, such as the Bunbury Basalt in southwestern Australia and the Comei igneous rocks in the eastern Tethyan Himalaya (Fig. 1). Some authors have argued that the widespread Early Cretaceous igneous rocks in the Tethyan Himalaya and southwestern Australia originated from the Kerguelen mantle plume, and hence the Kerguelen mantle plume played an important role in the breakup of eastern Gondwana^4,6,8–10^. However, some authors have proposed that these Early Cretaceous igneous rocks were partial melting of an asthenospheric mantle and that the breakup of eastern Gondwana was caused by passive rifting^12,13^. The reason for these contentious issues can be attributed to several factors: (1) the small volume (~ 11,000 km^3^) of the Comei-Bunbury LIP is incompatible with the typical mantle plume models^14^; and (2) the large distance (~ 1,000 km) between the Bunbury Basalt and the Kerguelen mantle plume^15,16^.

Igneous rocks associated with the Kerguelen mantle plume have been widely disseminated from their initial sites of emplacement due to the movements of the Antarctic, Australian, and Indian Plates^9,17–19^. Tethyan Himalaya was located at the northern margin of India, and thus the Tethyan Himalaya igneous rocks are crucial to understanding the relationship between the Kerguelen mantle plume and the breakup of eastern Gondwana. The close temporal coincidence between the Bunbury Basalt and the Comei igneous rocks (ca.132 Ma ago) prompted Zhu et al.^4^ to propose that the Comei-Bunbury LIP was probably caused by the Kerguelen mantle plume and that the Kerguelen mantle plume played a vital role in the breakup of eastern Gondwana. However, geochronological and geochemical results from the Charong Dolerites (ca.142 Ma) in the central Tethyan Himalayan indicate that the breakup of eastern Gondwana was caused by passive rifting rather than a mantle plume^13^. Notably, significant tectonic shortening has occurred due to the India-Asia collision since the emplacement of the LIP. As a result, the predicted size of the Comei LIP (> 40,000 km^2^) likely exceeds the LIP categorization (> 100,000 km^2^)^4^. However, some authors considered that the volume of the Comei-Bunbury LIP (~ 11,000 km^3^) is significantly smaller than that of typical mantle plume models (> 100,000 km^3^)^14,20^.

In the Tethyan Himalaya, the extensively dispersed igneous rocks from the Late Jurassic and Early Cretaceous are thought to be products of the Kerguelen mantle plume. Previous research primarily concentrated on the Sangxiu, Lakang, and Weimei formations^4,6,9,10,19,21–23^, with just a few studies focusing on the Zhela Formation^6,19,24^. Fossils identified in the Zhela Formation indicated a middle Jurassic age (1: 250,000 scale Longzi Country regional geological survey report (H46C004002), 2005). However, detrital zircon U–Pb geochronological data suggested that the Zhela Formation was deposited during the Early Cretaceous^24^. In this study, we provide new zircon U–Pb geochronological data of the Zhela Formation volcanic rocks from the eastern Tethyan Himalaya. Combining geochronological, geochemical, and palaeomagnetic data from the contemporary igneous rocks of the Tethyan Himalaya, we further discuss the initial activities of the Kerguelen mantle plume and the breakup of eastern Gondwana.

### Geological setting and samples

**Figure 2:**
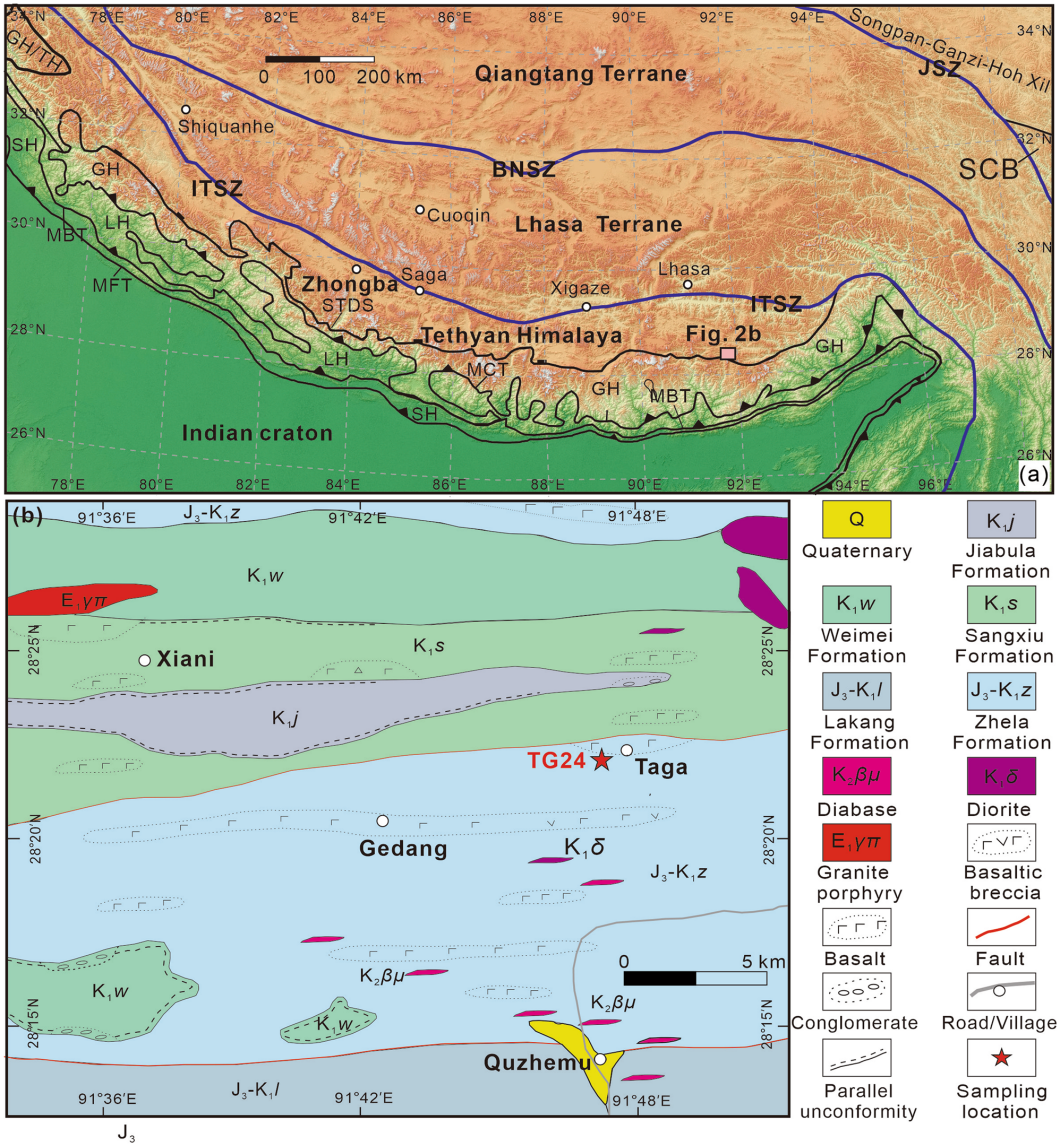
(**a**) Tectonic sketch map of the Himalayan belt and adjacent areas (after Yang et al.^21^). QGIS software (https://www.qgis.org/en/site/) and the Global Digital Elevation Model V003 data set from NASA Earth Data (http://search.earthdata.nasa.gov/search) were used to create the map. Abbreviations: SCB, South China Block; GH, Greater Himalaya; LH, Lesser Himalaya; SH, Sub-Himalaya; GH/TH, Tethyan Himalayan sequence in depositional contact with the underlying Greater Himalayan crystalline complex; JSZ, Jinsha suture zone; BNSZ, Bangong–Nujiang suture zone; ITSZ, Indus–Yarlung Tsangpo suture zone; MFT, Main Frontal Thrust; MBT, Main Boundary Thrust; MCT, Main Central Thrust; STDS, South Tibet detachment system. (**b**) Simplified geological map of the Taga area.

The Himalayan orogen is located at the northern margin of the Indian plate, which is well-known as an active tectonic belt due to the continuous northern movement of the Indian plate. It was divided into four tectonic units including the Tethyan Himalaya, the Greater Himalaya, the Lesser Himalaya, and the sub-Himalaya, from north to south^25^. These tectonic units within the Himalayan orogen are separated by the South Tibetan detachment system, the Main Central thrust, and the Main Boundary thrust from north to south (Fig. 2a). The Tethyan Himalaya, which was located at the northern margin of India, is comprised by Ordovician to Mesozoic sedimentary rocks, partially interlayered with Paleozoic and Mesozoic igneous rocks^25,26^.

The research area is located in the eastern Tethyan Himalaya where Upper Jurassic and Lower Cretaceous strata are well exposed (Fig. 2b). The Upper Jurassic and Lower Cretaceous strata mainly consist of the Zhela, Weimei, Sangxiu, Jiabula, and the Lakang formations. The Weimei Formation, which was deposited parallelly, unconformably underlies the Sangxiu Formation and conformably overlies the Zhela Formation. The contact relationship between the Sangxiu and Jiabula formations is a parallel unconformity.

**Figure 3:**
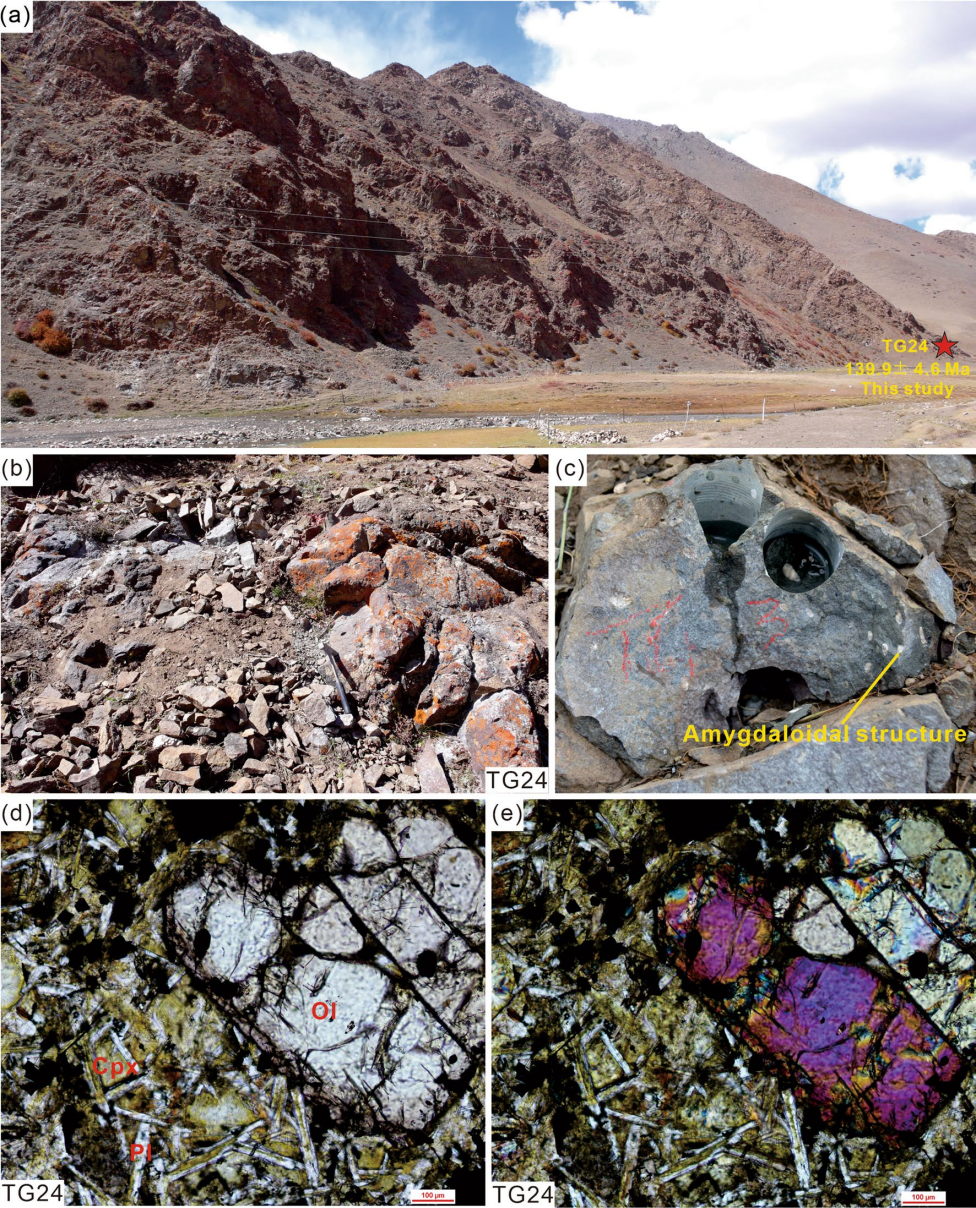
(**a**–**c**) Photographs showing field outcrop and (**d**, **e**) photomicrographs of the Zhela Formation volcanic rock (sample TG24) under plane-polarized light and cross-polarized light respectively. Abbreviations: Ol, olivine; Cpx, clinopyroxene; Pl, plagioclase.

The Zhela Formation is mainly composed of basalts and dacites interbedded with thin sandstones, slates, and limestones. It is assigned a Middle Jurassic age in the 1: 250,000 scale Longzi Country regional geological survey report (H46C004002, 2005). The Weimei Formation can be subdivided into two members. Member I consists of quartz sandstones, silty slates interbedded with silty metamorphic. Member II includes sericite silty slates interbedded with thin silt sandstones and sand-clastic limestones. The Sangxiu Formation also can be subdivided into two members. Member I consists of silt slates interbedded with sandstones, basalt, and conglomerate. Member II includes sericite silt slate interbedded with sandstones, basalt, and rhyolite. Zircon U–Pb geochronological results reveal that the Sangxiu Formation volcanic rocks formed during ca.136–124 Ma^4,22^. The Jiabula Formation mainly consists of quartz sandstone, gray siltstone, bioclastic siliceous rocks, shales, and a small amount of basalt interlayer. The Lakang Formation can be also subdivided into two members. Member I includes basalt interbedded with sedimentary rocks. Member II consists of silt slate, siltstone, and limestone. One fresh sample (TG24) was collected from the Zhela Formation volcanic rocks for zircon SHRIMP U–Pb dating (Fig. 3). Some of the outcrops show apparent amygdaloidal structure (Fig. 3c). The sample is basalt with a characteristic ophitic texture (Fig. 3d,e). The phenocrysts primarily consist of olivine and clinopyroxene. The groundmass mainly includes plagioclase and clinopyroxene.

## Results and discussion

### The ages of the sampling strata

**Figure 4:**
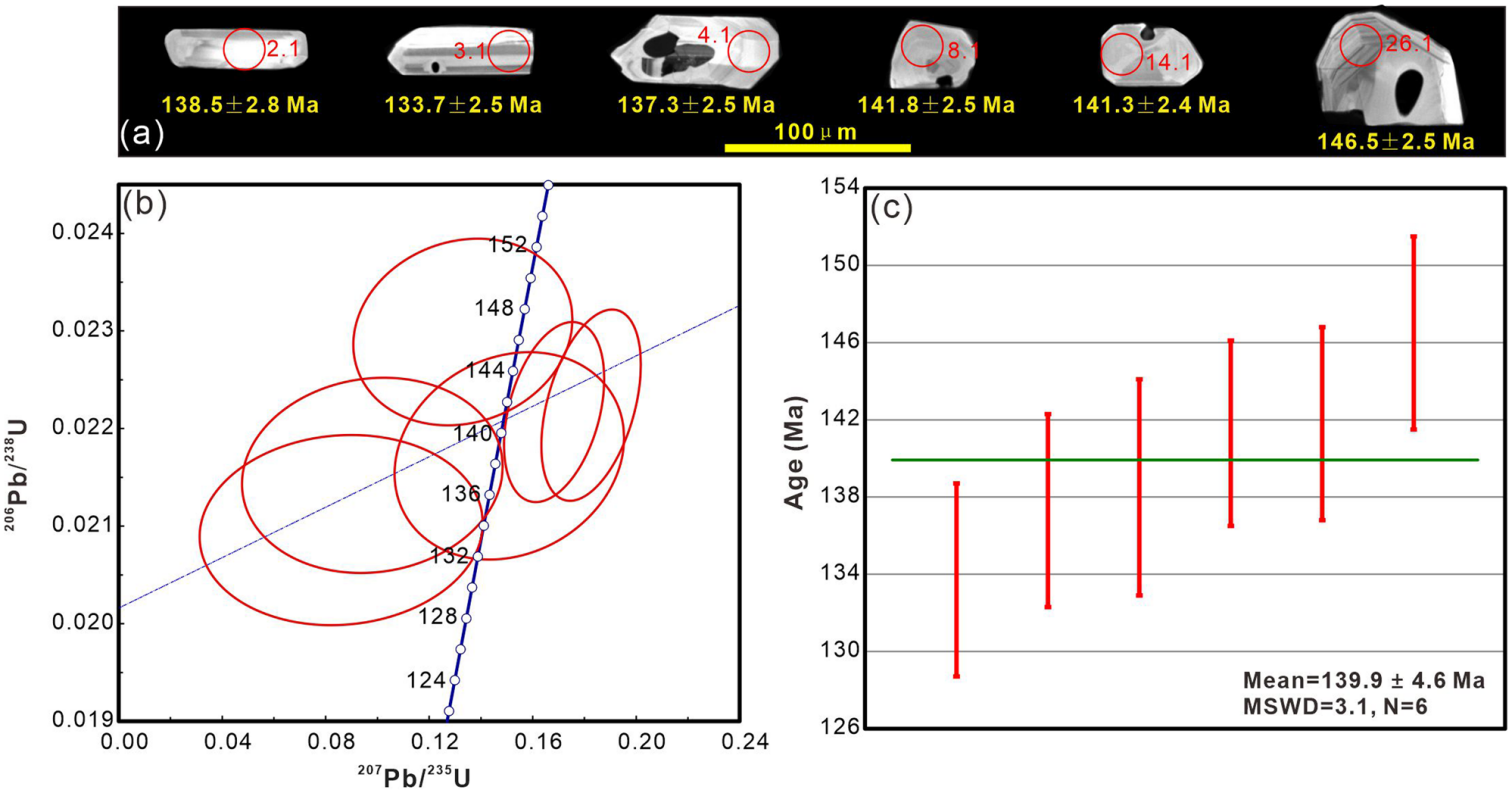
(**a**) Cathodoluminescence images of representative zircon grains and corresponding younger ^206^Pb/^238^U ages of the analyzed spots. (**b**) U–Pb concordia diagram of zircon grains. (**c**) Bar plot shows the weighted mean ^206^Pb/^238^U ages.

The zircons are subhedral (40–140 μm long, 20–60 μm wide) with weak oscillatory zoning (Fig. 4a and Supplementary Fig. S1). These characteristics, together with the fact that the Th/U ratios (0.11–1.35; Table 1) are obviously greater than the metamorphic zircon ratio (usually < 0.1), suggest that the zircons are of magmatic origin. Zircon SHRIMP U–Pb dating revealed a range of dates (Table 1), implying that these zircons came from various sources. The weighted mean ^206^Pb/^238^U ages of the youngest population are interpreted as the formation time of the volcanic rocks. The sample TG24 yielded ^206^Pb/^238^U ages ranging from 133.7 ± 2.5 Ma to 146.5 ± 2.5 Ma with a weighted average age of 139.9 ± 4.6 Ma (Fig. 4), which indicates that the sampled Zhela Formation volcanic rocks erupted during the Early Cretaceous. Other analyses on zircons yielded older ^206^Pb/^238^U ages ranging from 149.0 ± 2.5 Ma to 3460.0 ± 45.0 Ma, indicating an inherited origin (Supplementary Fig. S1).

**Table 1:** Zircon SHRIMP U–Pb ages for sample TG24 in the Taga area of the eastern Tethyan Himalaya.

Spot	^206^Pb_c_	U	Th	Th/U	^206^Pb*	^207^Pb*/^235^U	± %	^206^Pb*/^238^U	± %	^206^Pb/^238^U
	(%)	(ppm)	(ppm)		(ppm)					age (Ma)
1.1	0.34	122	146	1.20	18.90	2.010	2.5	0.1801	1.6	1068.0 ± 16.0
2.1	0.45	73	53	0.73	1.37	0.151	12.0	0.0217	2.0	138.5 ± 2.8
3.1	2.52	231	127	0.55	4.27	0.086	26.0	0.0210	1.9	133.7 ± 2.5
4.1	2.55	160	84	0.53	3.04	0.098	21.0	0.0215	1.9	137.3 ± 2.5
5.1	0.38	70	44	0.63	10.80	1.864	3.1	0.1784	1.7	1058.0 ± 16.0
6.1	0.13	56	56	1.00	1.73	0.261	7.2	0.0357	1.9	226.0 ± 4.3
7.1	0.34	57	26	0.46	9.65	2.043	3.4	0.1955	1.7	1151.0 ± 18.0
8.1	–	100	66	0.66	1.87	0.183	4.3	0.0222	1.8	141.8 ± 2.5
9.1	0.73	138	186	1.35	11.30	0.700	5.0	0.0944	1.6	581.4 ± 9.1
10.1	0.10	57	40	0.70	34.70	28.840	1.8	0.7100	1.7	3460.0 ± 45.0
11.1	0.39	184	240	1.30	13.20	0.625	3.4	0.0833	1.6	515.8 ± 7.7
12.1	3.78	96	57	0.59	3.20	0.174	28.0	0.0375	2.0	237.4 ± 4.7
13.1	0.02	312	283	0.91	49.40	1.942	1.7	0.1846	1.5	1092.0 ± 15.0
14.1	–	130	114	0.88	2.47	0.168	4.7	0.0222	1.7	141.3 ± 2.4
15.1	0.17	92	84	0.91	15.90	2.211	2.2	0.2014	1.6	1183.0 ± 18.0
16.1	0.08	238	27	0.11	38.00	1.938	1.8	0.1857	1.5	1098.0 ± 15.0
17.1	0.12	236	207	0.88	31.60	1.490	2.0	0.1554	1.5	931.0 ± 13.0
18.1	0.19	39	30	0.77	6.46	2.061	3.0	0.1924	1.9	1134.0 ± 20.0
19.1*	30.50	154	101	0.66	5.33			0.0280	23.0	178.0 ± 40.0
20.1	0.92	105	53	0.50	5.93	0.439	8.7	0.0649	1.8	405.3 ± 6.9
20.2	0.82	257	211	0.82	7.74	0.207	5.4	0.0347	1.6	220.1 ± 3.4
21.1	0.12	297	116	0.39	14.80	0.422	2.6	0.0578	1.5	362.3 ± 5.3
22.1	0.13	207	198	0.96	16.50	0.749	2.4	0.0927	1.5	571.3 ± 8.3
23.1	–	93	68	0.73	13.10	1.694	2.3	0.1651	1.6	985.0 ± 15.0
24.1	1.62	191	205	1.07	3.91	0.120	12.0	0.0234	1.7	149.0 ± 2.5
25.1	0.01	179	69	0.39	50.10	4.861	1.7	0.3260	1.5	1819.0 ± 24.0
26.1	1.03	199	190	0.95	3.98	0.133	13.0	0.0230	1.7	146.5 ± 2.5
27.1	0.03	314	56	0.18	42.30	1.580	2.0	0.1571	1.8	940.0 ± 16.0

Pb_c_ indicates common Pb; Pb* indicates radiogenic Pb; Errors are 1σ; Spot ID with * are discarded because of its higher common Pb.

Reliable chronological constraints for the sampling strata are vital for regional stratigraphic correlation and division. However, because of the intricate nature of the Tibetan Plateau, numerous layers have been assigned inaccurate ages^19,27,28^. For example, zircon SHRIMP U–Pb dating indicates that the Zhela Formation and Weimei Formation volcanic rocks of the Luozha area in the Tethyan Himalaya erupted during ca. 138–135 Ma^19^, not the Middle and Late Jurassic as given by the 1:250,000 scale Luozha regional geological report (H46C004001, 2002). Zircon LA-ICP-MS U–Pb dating indicates that the Risong Formation red beds and volcanic rocks of the Wuma area in the Lhasa terrane formed during the ca.120–106 Ma^28^, not the Late Jurassic as given by the 1:250,000 scale Wuma regional geological report (I44C004004, 2006).

Fossils identified in the Zhela Formation include bivalves (*Costamussium zandaensis*—*Quenstedtia xizang ensis* assemblages), ammonoids (*Dolikephalites*—*Indocephalites* and *Dorsetensia-Garantiana* assemblages) and belemnites (*Holcobelus cf. biainvillei*—*Hastites* and *Aractites longissima*—*Salpigotheuthis* assemblages), and are indicative of the Middle Jurassic by the 1:250,000 scale Longzi Country regional geological survey report (H46C004002, 2005). Our new zircon SHRIMP U–Pb dating and recent zircon LA-ICP-MS U–Pb ages (147.1 ± 2.5 Ma)^29^ from the same sampling region, however, show that the Zhela Formation volcanic rocks of the Taga area erupted during ca. 147–140 Ma, not during the Middle Jurassic. Our updated chronological results are broadly in agreement with the youngest detrital zircons ages of ca.127–138 Ma from the same region reported by Jiao et al.^24^ and the ages of ca. 135 Ma from the Luozha area reported by Bian et al.^19^. Furthermore, our chronological results are also supported by the bimodal magmatism (144–140 Ma) recently identified in the Taga area by Zhang et al.^30^.

### The volume of the Comei-Bunbury LIP

LIPs are usually characterized by the magmatic provinces with areal extents > 100,000 km^2^ and igneous volumes > 100,000 km^3^ based on Bryan and Ernst^1^. Despite the fact that palaeomagnetic data show that the Comei-Bunbury LIP is positioned in the heart of the Kerguelen mantle plume (see the following section), one essential piece of evidence that must be addressed is if the area and volume of the Comei-Bunbury igneous rocks are comparable to the LIPs. According to Coffin^8^, the area and volume of the Bunbury Basalt are ~ 100,000–1000,000 km^2^ and ~ 1,000 km^3^, respectively. In addition, the area and volume of the Comei igneous rocks are ~ 40,000 km^2^ based on Zhu et al.^4^ and ~ 10,000 km^3^ based on Liu et al.^14^, respectively. Although the area matches the LIPs, the small volume (~ 11,000 km^3^) of the Comei-Bunbury igneous rocks is incompatible with the typical mantle plume models^14^.

Notably, geochronological and geochemical results indicate that the Abor volcanic rocks from Eastern Himalayan Syntaxis in northeastern India^31^, the volcanic rocks from the Naturaliste Plateau, the Wallaby Plateau, and the Mentelle Bassin in southwestern Australia^32–34^ have geochemical and geochronological similarities to the Comei-Bunbury igneous rocks. The Abor volcanic rocks have an areal extent of ~ 2,500 km^2^ with an average thickness of 500 m^35^. The calculated volume of the Abor volcanic rocks is ~ 1,250 km^3^. The Early Cretaceous volcanic rocks from the Naturaliste Plateau (~ 90,000 km^2^)^34^, the Wallaby Plateau (~ 70,000 km^2^)^33^, and the Mentelle Bassin (~ 44,000 km^2^)^32^ have an areal extent of ~ 204,000 km^2^. Assuming an average thickness of 500–1000 m, the minimum volume of those volcanic rocks is 102,000 km^3^. Totally, the calculated volume of the Comei-Bunbury igneous rocks is ~ 114,250 km^3^. Therefore, the volume of Comei-Bunbury igneous rocks is compatible with the LIPs, and thus the typical mantle plume models.

### The palaeolatitudes of the Comei-Bunbury LIP

Because of the movements of the Indian, Australian, and Antarctic plates, igneous rocks associated with the Kerguelen mantle plume have been widely scattered from their initial positions of emplacement. Understanding the spatial interaction of these igneous rocks with the Kerguelen plume mantle requires determining their initial positions of emplacement. Palaeomagnetism is the only way to quantify the palaeolatitude of the plate^36^ and is thus essential to constrain the erupted position of these igneous rocks. Table 2 lists the reliable latest Jurassic to Early Cretaceous palaeomagnetic data from the Comei-Bunbury igneous rocks. Notably, based on the 1:250,000 Longzi regional geological survey report (H46C004002, 2004), Yang et al.^21^ assigned an Early Cretaceous date (134–131 Ma) to the Lakang Formation lava flows in the Cona area in the eastern Tethyan Himalaya. According to recent zircon LA-ICP-MS and SHRIMP U–Pb geochronological data, the Lakang Formation lava flows erupted at ca. 147–141 Ma^10,19,23^. The Abor volcanic rocks of the northeastern Indian craton were given an Early Permian date by Ali et al.^37^. New zircon LA-ICP-MS U–Pb analyses, however, have shown that the Abor volcanic rocks erupted at ca.133–131 Ma^31^. Additionally, based on K/Ar dating performed by McDougall and Wellman^38^, Schmidt^39^ gave a Late Cretaceous age to the Bunbury Basalt of southern Australia. According to new ^40^Ar/^39^Ar analytical results, the Bunbury Basalt erupted at ca.137–130 Ma^12^. This study made use of these fresh geochronological findings.

**Table 2:** Summary of the Late Jurassic-Early Cretaceous palaeomagnetic results from the Tethyan Himalaya, Indian craton, and Australia.

ID	Lithology	Area	Slat	Slon	Age	Plat	Plon	A_95_(dp/dm)	Palaeolat	References
			(°N)	(°E)	(Ma)	(°N)	(°E)	(°)	(°S)	
Tethyan Himalaya										
TG	Volc	Taga	28.4	91.8	ca.147–140	− 30.5	324.9	11.8	44.1 ± 11.8	^29^
LK	Volc	Cuona	28.1	92.4	ca.147–141	− 26.8	315.2	5.7	52.5 ± 5.7	^10,21,23^
ZD	Volc	Zhuode	28.9	91.3	ca.138–135	0.9	293.4	7.0	53.5 ± 7.0	^19^
SX	Volc	Langkazi	28.8	91.3	ca.135–124	− 5.9	308.0	6.1	45.3 ± 6.1	^22^
ZY	Volc	Langkazi	28.7	91.1	ca.134–133	6.3	308.6	9.1	39.7 ± 9.1	^41^
MC	Volc + Lim	Cona	28.1	92.4	ca.137–125	22.0	266.7	7.6	39.6 ± 7.6	^40^
Indian craton										
Ab	Volc	Siang valley	28.3	95.1	ca.133–131	− 24.6	313.6	8.6/10.2	55.5 ± 8.6	^31,37^
Australia										
BB	Volc	Bunbury	− 33.8	115.6	ca.137–130	− 50.0	163.0	4.0	52.0 ± 4.0	^12,39^

ID, palaeopoles abbreviation used in the text; Volc, volcanic rocks; Lim, Limestone; Slat (Slon), latitude (longitude) of sites; Plat (Plon), latitude (longitude) of poles; A_95_, 95% confidence range; dp/dm, semi-axes of elliptical error of the pole at a probability of 95%; Palaeolat, palaeolatitude calculated for the reference point located at their studied area.

The palaeopole (TG^29^) obtained from the Zhela Formation volcanic rocks in the Taga area of the eastern Tethyan Himalaya is well constrained by our new zircon U–Pb geochronology and yielded a palaeolatitude of 44.1° ± 11.8°S at ca.147–140 Ma for the reference point (28.4°N, 91.8°E) (Table 2). Furthermore, five palaeopoles obtained from the Cona (LK^21^ and MC^40^), Zhuode (ZD^19^), and Langkazi (SX^22^ and ZY^41^) areas of the eastern Tethyan Himalaya yielded palaeolatitudes of 52.5 ± 5.7°S at ca.147–141 Ma, 39.6 ± 7.6°S at ca.137–125 Ma, 53.5 ± 7.0°S at ca.138–135 Ma, 45.3 ± 6.1°S at ca.135–124 Ma, and 39.7 ± 9.1°S at ca.134–133 Ma for the reference point located at their studied area, respectively (Table 2). The palaeolatitude of the Taga area is identical to the palaeolatitudes obtained from the Cona, Zhuode, and Langkazi areas within 95% confidence limits, indicating that the eastern Tethyan Himalaya was located at ~ 39.6–53.5°S during ca.147–124 Ma. Similarly, the palaeopole (Ab^37^) obtained from the Abor volcanic rocks in the northeastern Indian craton yielded a palaeolatitude of 55.5 ± 8.6°S at ca.133–131 Ma for the reference point (28.3°N, 95.1°E) (Table 2). The palaeopole (BB^39^) obtained from the Bunbury Basalt in southwestern Australia yielded a palaeolatitude of 52.0 ± 4.0°S at ca.137–130 for the reference point (33.8°S, 115.6°E) (Table 2). These palaeomagnetic results indicate that the Comei-Bunbury igneous rocks, which are presently located at 28.1–28.9°N for the eastern Tethyan Himalaya, at 28.3°N for the northeastern Indian craton, and at 33.3–34.3°S for southwestern Australia, originally erupted at ~ 39.6–55.5°S. According to the hybrid reference frames described by Torsvik et al.^42^, the eruption center of the reconstructed Kerguelen mantle plume LIPs was located at ~ 41.6–52.3°S. The palaeolatitudes of the reconstructed Kerguelen mantle plume LIPs are identical to the palaeolatitudes of the Comei-Bunbury LIP (~ 39.6–55.5°S), indicating that the Comei-Bunbury igneous rocks came from the Kerguelen mantle plume.

### Implications for the breakup of eastern Gondwana

Because of the small volume of the Comei-Bunbury igneous rocks^13,14^, the large distance between the Bunbury Basalt and the Kerguelen mantle plume^15,16^, the relationship between the Comei-Bunbury igneous rocks and the Kerguelen mantle plume is still debated. Geochronological and geochemical results from the mafic rocks in the Taga area show that these mafic rocks erupted during ca. 144–140 Ma and that bimodal magmatism was identified as a response to early Kerguelen plume mantle activity^30^. Consider the similarity in sample area and age between the mafic rocks reported by Zhang et al.^30^ and the basalt investigated in this study, implying that they originated from the same source, the Kerguelen mantle plume. This is consistent with the geochronological and geochemical results from the Zhela formation volcanic rocks in the Zhuode area of the eastern Tethyan Himalaya^6^. The Sangxiu, Lakang, Zhela, and Weimei formations volcanic rocks in the eastern Tethyan Himalaya showed no obvious Eu anomalies and shared geochemical characteristics that high contents of TiO_2_, highly fractionated in light rare earth elements and heavy rare earth elements, and indicated similarities to the alkali basalts originated from the Kerguelen mantle plume^6,9,10^. This, together with the recalculated volume of the Comei-Bunbury igneous rocks and the spatial relationships between the Comei-Bunbury igneous rocks and the Kerguelen mantle plume as we mentioned above, indicate that the Comei-Bunbury igneous rocks originated from the Kerguelen mantle plume.

**Figure 5:**
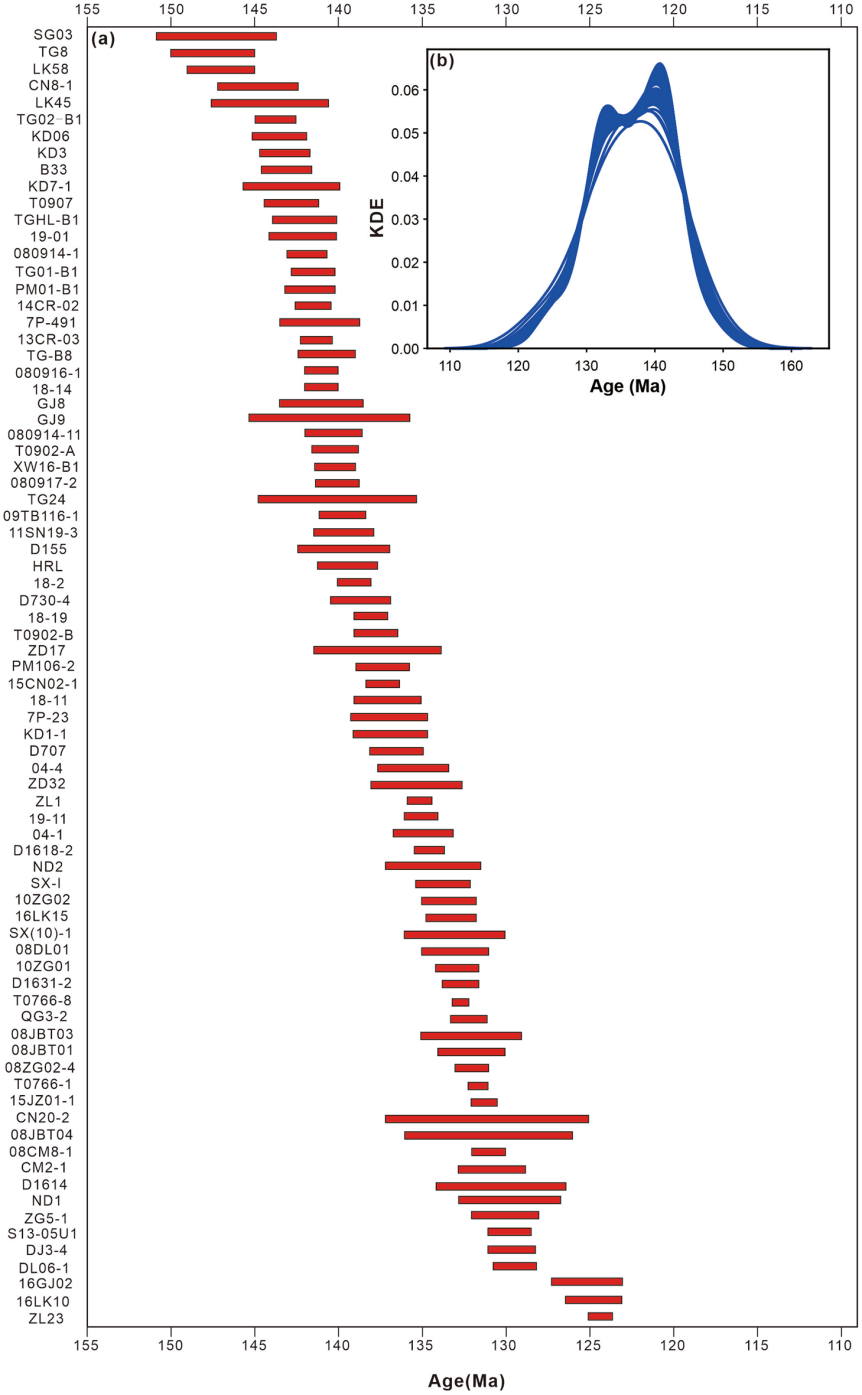
(**a**) Zircon U–Pb ages for samples of the Comei LIP in the Tethyan Himalaya. See Supplementary Table S1 for data compilation and references. (**b**) Simulation of kernel density estimate variance using 100 trials where dates are randomly selected for each of the ages in Supplementary Table S1 using their ages and uncertainties^56^. Two potential peaks can be identified at ca. 141 Ma and ca. 133 Ma in 97 trials in the kernel density estimate plot. Abbreviations: KDE, kernel density estimate.

There is no agreement on whether the breakup of eastern Gondwana was caused by a mantle plume^9,11,20^ or passive rifting^13^. The ages of the volcanism attributed to the Kerguelen mantle plume in the Tethyan Himalaya range from ca.147 Ma to ca.124 Ma (Supplementary Table S1 and Fig. 5)^4,6,9–11,13,14,19,22,23,29,30,41,43–55^ with two peaks at approximately 141 Ma and 133 Ma^56^ (Fig. 5b). The Comei igneous rocks' eruption characteristics also match those of LIPs with overall eruptive durations of around 50 Myr, including vigorous eruptive pulse(s) of approximately 1–5 Myr, during which a significant proportion (> 75%) of the entire volume of the LIP is emplaced^1^.

**Figure 6:**
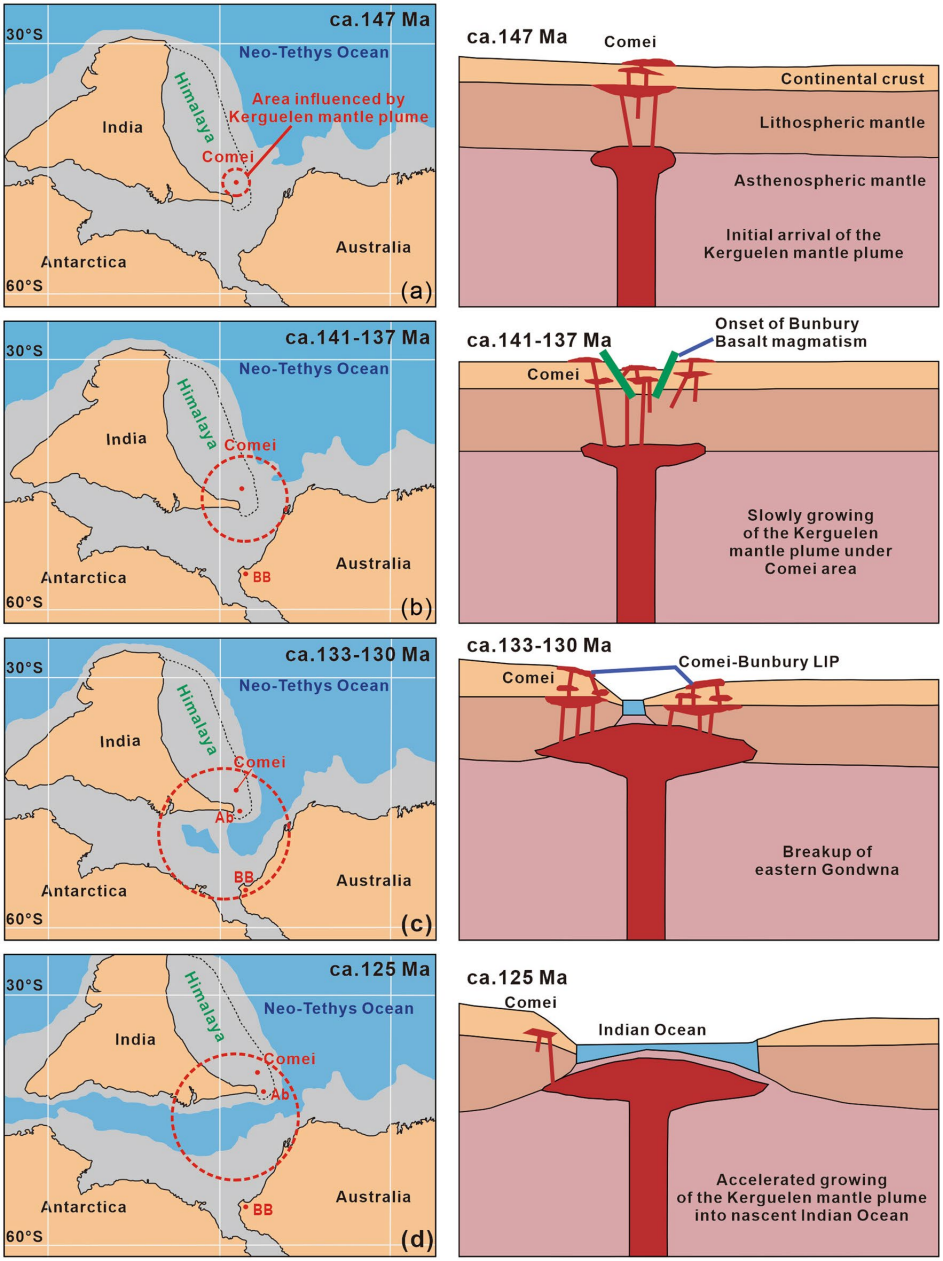
(**a**–**d**) Plate and plume reconstructions demonstrating the breakup of the Indian plate from the eastern Gondwana (based on this study and the relative plate reconstruction of Mattews et al.^57^ using Gplates^58^ (https://www.gplates.org/)) and tectonic–magmatic evolution related to the Kerguelen mantle plume (after Wang et al.^11^) at 147 Ma, 141–137 Ma, 133–130 Ma, and 125 Ma, respectively. Abbreviations: BB, Bunbury Basalt; Ab, Abor volcanic rocks.

According to zircon SHRIMP U–Pb dating and geochemical study from the Lakang Formation volcanic rocks in the eastern Tethyan Himalaya, the Kerguelen mantle plume began in the Late Jurassic (ca.147 Ma), and it played an essential part in the breakup of eastern Gondwana^10^. Furthermore, gabbro-diabase and gabbro samples from the Rongbu area in the eastern Tethyan Himalaya share geochemical characteristics with the volcanic rocks of the Lakang and Sangxiu formations, which both formed on the continental margin in a significantly extensional rift zone^43^. These findings suggest that the Kerguelen mantle plume’s activity began at ca. 147 Ma and that eastern Gondwana first disintegrated and dispersed at that time. However, no volcanic rocks older than 137 Ma have been discovered in southwestern Australia. This might be explained by the fact that the eastern Tethyan Himalaya was situated in the center of the plume head (Fig. 6a^11,30,57,58^).

With the breakup of the eastern Gondwana and the growth of mantle plumes, the Indian plate started moving northward after ca.140 Ma^59^, and the oldest oceanic crust (ca.140 Ma) off western Australia grew younger to the west^60^. Furthermore, marine magnetic anomalies for the west Australian margin suggested that the mid-ocean ridge that separated the Indian plate from the Australian-Antarctic plate began at ~ 136 Ma. According to ^40^Ar/^39^Ar geochronological and whole-rock geochemical studies, the Bunbury Basalt erupted in three separate phases (ca. 137 Ma, ca. 133 Ma, and ca. 130 Ma) and shared geochemical similarities with the Kerguelen mantle plume-products^12^. Olierook et al.^33^ suggested that the Bunbury Basalt erupted in the last two phases originated from the same flow. Therefore, the primary eruption period of the Bunbury Basalt is generally consistent with that of the Comei igneous rocks, both of which may have peaked during 141–137 Ma and 133–130 Ma (Fig. 6b,c).

The eastern Tethyan Himalaya advanced to the edge of the mantle plume when the Indian plate moved northward, generating the ca.125 Ma OIB magmatic rocks^11^. The bimodal magnetic rocks (118–115 Ma) and N-MORB-like mafic rocks (ca.120 Ma) discovered in the eastern Tethyan Himalaya are the result of an extensional environment and are not the products of Kerguelen mantle plumes^45,61^. These findings, together with the fact that the Australian-Antarctic plate maintained a relatively stable palaeolatitude during 140–120 Ma^59^, suggest that the Indian plate separated completely from the eastern Gondwana before ca.125 Ma (Fig. 6d).

## Conclusions

We have presented new zircon SHRIMP U–Pb geochronological results from the Zhela Formation volcanic rocks in the Tethyan Himalaya. Our new results, together with previous geochronological, geochemical, and palaeomagnetic data from the Comei-Bunbury LIP, led us to draw the following conclusions: (1) the studied Zhela Formation volcanic rocks formed during ca.147–140 Ma, rather than the Middle Jurassic as assigned by the 1:250,000 scale Longzi regional geological survey report; (2) the calculated volume of the Comei-Bunbury igneous rocks is ~ 114,250 km^3^, which is compatible with the LIPs, and thus the typical mantle plume models; (3) the volcanism attributed to the Kerguelen mantle plume in the Tethyan Himalaya range from ca.147 Ma to ca.124 Ma, with two peaks at approximately 141 Ma and 133 Ma. (4) the palaeolatitudes of the Comei-Bunbury LIP are identical to the palaeolatitudes of the reconstructed Kerguelen mantle plume LIPs; (5) the Comei-Bunbury igneous rocks originated from the Kerguelen mantle plume, and the Kerguelen mantle plume contributed significantly to the breakup of eastern Gondwana; (6) eastern Gondwana first disintegrated and dispersed at ca.147 Ma, and the Indian plate separated completely from the eastern Gondwana before ca.125 Ma.

## Methods

One fresh block sample was collected from the Zhela Formation volcanic rocks near Taga village located at ~ 45 km west of Longzi town (28°22′11.4″N, 91°47′12.6″E) in the eastern Tethyan Himalaya. Zircons for SHRIMP U–Pb dating were extracted by magnetic cleaning and heavy mineral separation from crushed samples and finally selected by hand-picking under the binoculars. The selected zircons were mounted onto an epoxy resin disc together with some standard zircon grains and then ground down and polished to expose their interiors. Transmission, reflected and cathodoluminescence images photographed by optical microscopy were used to check their internal structures for subsequent SHRIMP U–Pb dating.

Zircon SHRIMP U–Pb dating was conducted at SHRIMP IIe at the Beijing SHRIMP Center, Institute of Geology, Chinese Academy of Geological Sciences, Beijing, China. Software packages Squid^62^ and Isoplot^63^ were used to process the data. During testing, the mass resolution was 5,000 (1% definition), the spot sizes were 25–30 μm, and the ion flow intensity of O_2_^-^ was 4 nA. The U and Th contents of the unidentified zircon particles were calibrated using a standard zircon sample M257. To ensure the precision and reliability of the experimental data, the standard TEMORA zircon grain calibration was carried out after every fourth analysis. The weighted mean ages are provided at the 95% confidence interval, while the uncertainties for individual analysis are quoted at the 1-sigma level.

### Supplementary Information

Supplementary Information.

## Data Availability

All data generated or analysed during this study are included in this published article [and its supplementary information files].
