# Supplementary material for: Geochronological results from the Zhela Formation volcanics of the Tethyan Himalaya and their implications for the breakup of eastern Gondwana
Source: Sci Rep. 2023 Nov 16;13:20035. doi: 10.1038/s41598-023-47268-5 (PMC10654728; doi:10.1038/s41598-023-47268-5)
Supplement: Supplementary file 1 — Supplementary Information. [file 41598_2023_47268_MOESM1_ESM.docx]

**Supplementary Information**

**Geochronological results from the Zhela Formation volcanics of the Tethyan Himalaya and their implications for the breakup of eastern Gondwana**

**Jiacheng Liang^1,2^****, Weiwei Bian^1*^, Xianwei Jiao^1,2^, Wenxiao Peng^1,2^, Jiahui Ma^1,2^, Suo Wang^1,2^, Yiming Ma^3^, Shihong Zhang^1,2^, Huaichun Wu^1^, Haiyan Li^1^, Yuruo Shi^4^, Tianshui Yang^1,2^**

*^1^ State Key Laboratory of Biogeology and Environmental Geology, China University of Geosciences, Beijing 100083, China*

*^2^ School of Earth Sciences and Resources, China University of Geosciences, Beijing 100083, China*

*^3^ School of Earth Sciences and Resources, China University of Geosciences, Wuhan 430074, China*

*^4^ Beijing SHRIMP Center, Institute of Geology, Chinese Academy of Geological Sciences, Beijing 100037, China*

Corresponding author: Weiwei Bian [(bianww@cugb.edu.cn)](mailto:(bianww@cugb.edu.cn))

**
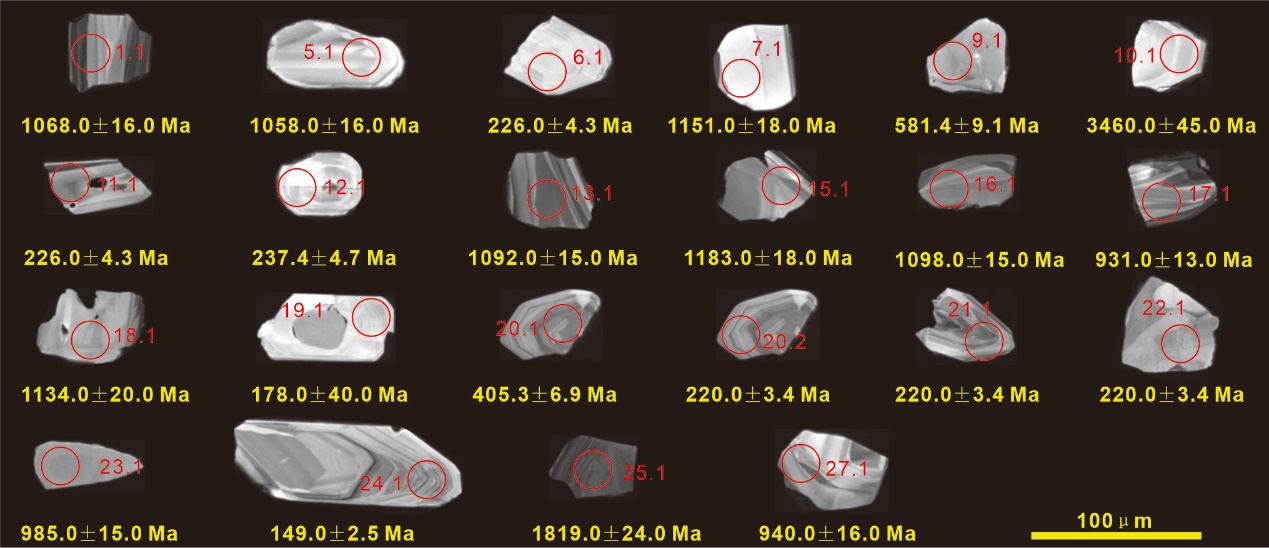
Figure S1.** Cathodoluminescence images of representative zircon grains and corresponding older ^206^Pb/^238^U ages of the analyzed spots.

**Supplementary Table S1**. Summary of zircon U-Pb ages for samples of the Comei LIP in the Tethyan Himalaya.

| Sample | Rock type | Area | Slat | Slon | Age | Technique | Reference |
| --- | --- | --- | --- | --- | --- | --- | --- |
|  |  |  | (°N) | (°E) | (Ma) |  |  |
| SG03 | Gabbro | Rongbu | 28.7 | 91.2 | 147.3±3.6 | U-Pb LA-ICP-MS | 43 |
| TG8 | Lava flows | Taga | 28.4 | 91.8 | 147.1±2.5 | U-Pb LA-ICP-MS | 29 |
| LK58 | Andesite | Cona | 28.1 | 92.4 | 147.0±2.0 | U-Pb SHRIMP | 10 |
| CN8-1 | Diabase | Cona | 28.1 | 92.0 | 144.7±2.4 | U-Pb SHRIMP | 9 |
| LK45 | Lava flows | Cona | 28.1 | 92.3 | 144.1±3.5 | U-Pb LA-ICP-MS | 19 |
| TG02-B1 | Dacite | Taga | 28.3 | 91.7 | 143.7±1.2 | U-Pb LA-ICP-MS | 30 |
| KD06 | Diabase | Cona | 28.6 | 92.4 | 143.5±1.6 | U-Pb LA-ICP-MS | 44 |
| KD3 | Diabase | Cona | 28.6 | 92.3 | 143.2±1.5 | U-Pb LA-ICP-MS | 44 |
| B33 | Dacite | Cona | 28.4 | 92.3 | 143.0±1.5 | U-Pb LA-ICP-MS | 44 |
| KD7-1 | Diabase | Cona | 28.6 | 92.4 | 142.8±2.9 | U-Pb LA-ICP-MS | 44 |
| T0907 | Diabase | Gyangze | - | - | 142.7±1.6 | U-Pb SHRIMP | 45 |
| TGHL-B1 | Gabbro | Taga | 28.3 | 91.7 | 142.1±1.9 | U-Pb LA-ICP-MS | 30 |
| 19-01 | Gabbro | Cona | 28.1 | 92.4 | 142.0±2.0 | U-Pb SHRIMP | 46 |
| 080914-1 | Volcanics | Yamzho Yumco | 28.8 | 90.7 | 141.8±1.2 | U-Pb SIMS | 47 |
| TG01-B1 | Dacite | Taga | 28.3 | 91.7 | 141.7±1.3 | U-Pb LA-ICP-MS | 30 |
| PM01-B1 | Diabase | Taga | 28.3 | 91.7 | 141.6±1.5 | U-Pb LA-ICP-MS | 30 |
| 14CR-02 | Diabase | Charong | 29.0 | 88.4 | 141.6±1.1 | U-Pb LA-ICP-MS | 13 |
| 7P-491 | Quartz monzonite | Cona | 28.4 | 92.4 | 141.2±2.4 | U-Pb LA-ICP-MS | 44 |
| 13CR-03 | Diabase | Charong | 29.0 | 88.4 | 141.1±0.9 | U-Pb LA-ICP-MS | 13 |
| TG-B8 | Diabase | Taga | 28.3 | 91.7 | 141.0±1.7 | U-Pb LA-ICP-MS | 30 |
| 080916-1 | Volcanics | Yamzho Yumco | 28.8 | 90.7 | 141.0±1.0 | U-Pb SIMS | 47 |
| 18-14 | Gabbro | Cona | 28.1 | 92.4 | 141.0±1.0 | U-Pb SHRIMP | 46 |
| GJ8 | Basalt | Cona | 28.1 | 92.3 | 140.7±2.4 | U-Pb SHRIMP | 23 |
| GJ9 | Basalt | Cona | 28.1 | 92.3 | 140.7±4.5 | U-Pb SHRIMP | 23 |
| 080914-11 | Volcanics | Yamzho Yumco | 28.8 | 90.7 | 140.6±1.7 | U-Pb SIMS | 47 |
| T0902-A | Diabase | Gyangze | - | - | 140.6±1.4 | U-Pb SHRIMP | 45 |
| XW16-B1 | Diabase | Taga | 28.3 | 91.7 | 140.4±1.2 | U-Pb LA-ICP-MS | 30 |
| 080917-2 | Volcanics | Yamzho Yumco | 28.8 | 90.7 | 140.0±1.3 | U-Pb SIMS | 47 |
| TG24 | Lava flows | Taga | 28.4 | 91.8 | 139.9±4.7 | U-Pb SHRIMP | This study |
| 09TB116-1 | Granite | Comei | 28.4 | 91.5 | 139.7±1.4 | U-Pb SIMS | 48 |
| 11SN19-3 | Granite | Comei | 28.4 | 91.5 | 139.6±1.8 | U-Pb LA-ICP-MS | 48 |
| D155 | Diabase | Comei | 28.5 | 91.9 | 139.6±2.7 | U-Pb LA-ICP-MS | 49 |
| HRL | Quartz monzonite | Cona | 28.3 | 91.8 | 139.5±1.8 | U-Pb LA-ICP-MS | 44 |
| 18-2 | Gabbro | Cona | 28.1 | 92.4 | 139.0±1.0 | U-Pb SHRIMP | 46 |
| D730-4 | Diabase | Comei | 28.4 | 92.1 | 138.6±1.8 | U-Pb LA-ICP-MS | 49 |
| 18-19 | Monzogranite | Cona | 28.1 | 92.4 | 138.0±1.0 | U-Pb SHRIMP | 46 |
| T0902-B | Diabase | Gyangze | - | - | 137.7±1.3 | U-Pb SHRIMP | 45 |
| ZD17 | Lava flows | Luozha | 28.9 | 91.3 | 137.6±3.8 | U-Pb SHRIMP | 19 |
| PM106-2 | Allgovite | Rongbu | 28.8 | 90.5 | 137.3±1.6 | U-Pb LA-ICP-MS | 43 |
| 15CN02-1 | Basalt | Cona | 28.2 | 91.8 | 137.3±1.0 | U-Pb LA-ICP-MS | 50 |
| 18-11 | Monzogranite | Cona | 28.1 | 92.4 | 137.0±2.0 | U-Pb SHRIMP | 46 |
| 7P-23 | Dacite | Cona | 28.4 | 92.4 | 137.0±2.3 | U-Pb LA-ICP-MS | 44 |
| KD1-1 | Granite | Cona | 28.5 | 92.4 | 136.9±2.2 | U-Pb LA-ICP-MS | 44 |
| D707 | Felsic intrusive | Comei | 28.4 | 92.0 | 136.5±1.6 | U-Pb LA-ICP-MS | 49 |
| 04-4 | Diabase | Yamzho Yumco | 29.1 | 90.4 | 135.5±2.1 | U-Pb SHRIMP | 51 |
| ZD32 | Lava flows | Luozha | 28.9 | 91.3 | 135.3±2.7 | U-Pb SHRIMP | 19 |
| ZL1 | Lava flows | Langkazi | 28.8 | 91.3 | 135.1±0.7 | U-Pb LA-ICP-MS | 22 |
| 19-11 | Monzogranite | Cona | 28.1 | 92.4 | 135.0±1.0 | U-Pb SHRIMP | 46 |
| 04-1 | Diabase | Yamzho Yumco | 28.9 | 90.4 | 134.9±1.8 | U-Pb SHRIMP | 51 |
| D1618-2 | Gabbro | Comei | 28.3 | 92.4 | 134.6±0.9 | U-Pb LA-ICP-MS | 14 |
| ND2 | Volcanics | Luozha | 28.9 | 91.3 | 134.2±2.8 | U-Pb SHRIMP | 6 |
| SX-I | Andesite | Langkazi | 28.7 | 91.1 | 133.7±1.6 | U-Pb LA-ICP-MS | 41 |
| 10ZG02 | Diorite | Cona | 28.7 | 91.6 | 133.4±1.6 | U-Pb LA-ICP-MS | 52 |
| 16LK15 | Gabbro | Cona | 28.1 | 92.5 | 133.3±1.5 | U-Pb SHRIMP | 11 |
| SX(10)-1 | Dacite | Rimova village | 28.7 | 90.7 | 133.0±3.0 | U-Pb SHRIMP | 53 |
| 08DL01 | Diabase | Dalong village | 28.8 | 90.4 | 133.0±2.0 | U-Pb LA-ICP-MS | 4 |
| 10ZG01 | Tonalite | Cona | 28.7 | 91.6 | 132.9±1.3 | U-Pb LA-ICP-MS | 52 |
| D1631-2 | Diabase | Comei | 28.3 | 92.4 | 132.7±1.1 | U-Pb LA-ICP-MS | 14 |
| T0766-8 | Diabase | Dala | 28.6 | 92.3 | 132.7±0.5 | U-Pb LA-ICP-MS | 54 |
| QG3-2 | Diabase | Qonggyai reservoir | 28.9 | 91.6 | 132.2±1.1 | U-Pb SHRIMP | 4 |
| 08JBT03 | Gabbro | Dongjia village | 28.7 | 90.8 | 132.0±3.0 | U-Pb LA-ICP-MS | 4 |
| 08JBT01 | Diabase | Dongjia village | 28.6 | 90.8 | 132.0±2.0 | U-Pb LA-ICP-MS | 4 |
| 08ZG02-4 | Gabbro | Chigu Tso | 28.6 | 91.7 | 132.0±1.0 | U-Pb LA-ICP-MS | 4 |
| T0766-1 | Diabase | Dala | 28.6 | 92.2 | 131.7±0.6 | U-Pb LA-ICP-MS | 54 |
| 15JZ01-1 | Diabase | Gyangze | 28.8 | 89.8 | 131.3±0.8 | U-Pb LA-ICP-MS | 50 |
| CN20-2 | Gabbro | Cona | 28.1 | 92.4 | 131.1±6.1 | U-Pb SHRIMP | 8 |
| 08JBT04 | Dacite | Rimova village | 28.7 | 90.7 | 131.0±5.0 | U-Pb LA-ICP-MS | 4 |
| 08CM8-1 | Gabbro | Comei | 28.5 | 91.4 | 131.0±1.0 | U-Pb LA-ICP-MS | 4 |
| CM2-1 | Diabase | Comei | 28.5 | 91.5 | 130.8±2.0 | U-Pb LA-ICP-MS | 4 |
| D1614 | Granodiorite | Comei | 28.3 | 92.4 | 130.2±3.9 | U-Pb LA-ICP-MS | 14 |
| ND1 | Volcanics | Luozha | 28.9 | 91.3 | 130.0±3.0 | U-Pb SHRIMP | 6 |
| ZG5-1 | Pyroxenite | Chigu Tso | 28.6 | 91.7 | 130.0±2.0 | U-Pb SHRIMP | 4 |
| S13-05U1 | Diabase | Zhongba | 29.7 | 83.7 | 129.8±1.3 | U-Pb LA-ICP-MS | 55 |
| DJ3-4 | Gabbro | Dongjia village | 28.7 | 90.8 | 129.7±1.4 | U-Pb SHRIMP | 4 |
| DL06-1 | Diabase | Dalong village | 28.8 | 90.4 | 129.5±1.3 | U-Pb SHRIMP | 4 |
| 16GJ02 | Gabbro | Cona | 28.0 | 92.3 | 125.1±2.1 | U-Pb SHRIMP | 11 |
| 16LK10 | Gabbro | Cona | 28.1 | 92.5 | 124.8±1.7 | U-Pb SHRIMP | 11 |
| ZL23 | Lava flows | Langkazi | 28.8 | 91.3 | 124.4±0.7 | U-Pb LA-ICP-MS | 22 |

*Notes:* Slat (Slon), latitude (Longitude) of samples.

**References**

1. Zhu, D. et al. The 132 Ma Comei-Bunbury large igneous province: Remnants identifi ed in present-day southeastern Tibet and southwestern Australia. *Geology* **37,** 583–586 (2009).
2. Peng, W. et al. Role of the Kerguelen mantle plume in breakup of eastern Gondwana: Evidence from early cretaceous volcanic rocks in the eastern Tethyan Himalaya. *Palaeogeogr. Palaeoclimatol. Palaeoecol.* **588,** 110823 (2022).
3. Zhu, D. et al. Petrogenesis of the earliest Early Cretaceous mafic rocks from the Cona area of the eastern Tethyan Himalaya in south Tibet: Interaction between the incubating Kerguelen plume and the eastern Greater India lithosphere? *Lithos* **100,** 147–173 (2008).
4. Shi, Y. et al. Zircon SHRIMP U-Pb age of Late Jurassic OIB-type volcanic rocks from the Tethyan Himalaya: constraints on the initial activity time of the Kerguelen mantle plume. *Acta Geochim.* **38,** 441–455 (2018).
5. Wang, Z. et al. Early activity of the Kerguelen Mantle plume: geochronology, geochemistry and Sr-Nd-Pb isotopes of mafic dykes and sills from the Tethyan Himalaya. *Int. Geol. Rev.* **65,** 512–526 (2023).
6. Zeng, Y. et al. Breakup of Eastern Gondwana as inferred from the Lower Cretaceous Charong Dolerites in the central Tethyan Himalaya, southern Tibet. *Palaeogeogr. Palaeoclimatol. Palaeoecol.* **515,** 70–82 (2019).
7. Liu, Z. et al. Petrogenesis of the Early Cretaceous Laguila bimodal intrusive rocks from the Tethyan Himalaya: Implications for the break-up of Eastern Gondwana. *Lithos* **236–237,** 190–202 (2015).
8. Bian, W. et al. Paleomagnetic and Geochronological results from the Zhela and Weimei Formations Lava Flows of the Eastern Tethyan Himalaya: New Insights into the Breakup of Eastern Gondwana. *J. Geophys. Res. Solid Earth* **124,** 44–64 (2019).
9. Ma, Y. et al. Early Cretaceous paleomagnetic and geochronologic results from the Tethyan Himalaya: Insights into the Neotethyan paleogeography and the India–Asia collision. *Sci. Rep.* **6,** 21605 (2016).
10. Hou, C. Zircon SHRIMP U-Pb Age and Geochemistry of the Lakang Formation Volcanic Rocks in Tethyan Himalaya. Master thesis, Beijing, China, China University of Geoscience (2017) (in Chinese with English Abstract).
11. Deng, J. A combined paleomagnetic and zircon U-Pb geochronological study on the Zhela Formation volcanic rocks in the Tethyan Himalaya. Master thesis, Beijing, China, China University of Geosciences (2021) (in Chinese with English abstract).
12. Zhang, Z. et al. The evolution of Kerguelen mantle plume and breakup of eastern Gondwana: New insights from multistage Cretaceous magmatism in the Tethyan Himalaya. *Gondwana Res.* **119,** 68–85 (2023).
13. Zhang, Y., Huang, B. & Zhao, Q. New paleomagnetic positive proof of the rigid or quasi-rigid Greater Indian plate during the Early Cretaceous. *Chin. Sci. Bull.* **64,** 2225–2244 (2019).
14. Ding, F., Gao, J. & Xu, K. Geochemistry, geochronology and geological significances of the basic dykes in Rongbu area, Soutern Tibet. *Acta Petrol. Sin.* **36,** 391–408 (2020) (in Chinese with English abstract).
15. Huang, Y. et al. Petrogenesis of the Early Cretaceous Kada igneous rocks from Tethyan Himalaya: Implications for initial break‐up of eastern Gondwana. *Geol. J.* **54,** 1294–1316 (2019).
16. Wang, Y. et al. Multiple phases of cretaceous mafic magmatism in the Gyangze-Kangma area, Tethyan Himalaya, southern Tibet. *Acta Petrol. Sin.* **32,** 3572–3596 (2016) (in Chinese with English abstract).
17. Tian, Y. et al. Early Cretaceous bimodal magmatism in the eastern Tethyan Himalayas, Tibet: Indicative of records on precursory continental rifting and initial breakup of eastern Gondwana. *Lithos* **324–324,** 699–715 (2019).
18. Liu, Y. et al. U-Pb Zircon Ages of Early Cretaceous Volcanic Rocks in the Tethyan Himalaya at Yangzuoyong Co Lake, Nagarze, Southern Tibet, and Implications for the Jurassic/Cretaceous Boundary. *Cretac. Res.* **40,** 90–101 (2013).
19. Ma, L., Kerr, A. C., Wang, Q., Jiang, Z. & Hu, W. Early Cretaceous (~140 Ma) aluminous A-type granites in the Tethyan Himalaya, Tibet: Products of crust-mantle interaction during lithospheric extension. *Lithos* **300–301,** 212–226 (2018).
20. Zhou, Q. et al. Petrogenesis of mafic and felsic rocks from the Comei large igneous province, South Tibet: Implications for the initial activity of the Kerguelen plume. *Geol. Soc. Am. Bull.* **130,** 811–824 (2018).
21. Chen, S., Fan, W., Shi, R., Xu, J. & Liu, Y. The Tethyan Himalaya Igneous Province: Early Melting Products of the Kerguelen Mantle Plume. *J. Petrol.* **62,** 1–22 (2021).
22. Jiang, S., Nie, F., Hu, P. & Liu, Y. An important spreading event of the Neo-Tethys Ocean during the Late Jurassic and Early Cretaceous: Evidence from zircon U-Pb SHRIMP dating on diabase in Nagarze, southern Tibet. *Acta Geol. Sin.* **80,** 522–527 (2006).
23. Xia, Y. et al. Intermediate rocks in the Comei large igneous provinces produced by amphibole crystallization of tholeiitic basaltic magma. *Lithos* **374–375,** 105731 (2020).
24. Zhu, D. et al. SHRIMP U-Pb zircon dating for the dacite of the Sangxiu Formation in the central segment of Tethyan Himalaya and its implications. *Chin. Sci. Bull.* **50,** 563–568 (2005).
25. Wang, Y. et al. Early Cretaceous high-Ti and low-Ti mafic magmatism in Southeastern Tibet: Insights into magmatic evolution of the Comei Large Igneous Province. *Lithos* **296–299,** 396–411 (2018).
26. Wei, Y., Liang, W., Shang, Y., Zhang, B. & Pan, W. Petrogenesis and tectonic implications of ∼130 Ma diabase dikes in the western Tethyan Himalaya (western Tibet). *J. Asian Earth Sci.* **143,** 236–248 (2017).
